# Effects of Mangiferin on LPS-Induced Inflammation and SARS-CoV-2 Viral Adsorption in Human Lung Cells

**DOI:** 10.3390/pharmaceutics14122845

**Published:** 2022-12-19

**Authors:** Mariarita Spampinato, Giuseppe Carota, Giuseppe Sferrazzo, Virginia Fuochi, Alfio Distefano, Simone Ronsisvalle, Federica Sipala, Rosario Giuffrida, Pio Maria Furneri, Michelino Di Rosa, Daniele Tibullo, Giovanni Li Volti, Ignazio Barbagallo

**Affiliations:** 1 Department of Biomedical and Biotechnological Sciences, University of Catania, Via S. Sofia 87, 95125 Catania, Italy; 2 Department of Drug and Health Sciences, University of Catania, Viale A. Doria 6, 95125 Catania, Italy

**Keywords:** COVID-19, *Mangifera indica* L., anti-inflammation, antiviral

## Abstract

The growing interest in natural bioactive molecules, as an approach to many pathological contexts, is widely justified by the necessity to overcome the disadvantageous benefit–risk ratio related to traditional therapies. Among them, mangiferin (MGF) shows promising beneficial properties such as antioxidant, anti-inflammatory, and immunomodulatory effects. In this study, we aimed to investigate the antioxidant and anti-inflammatory properties of MGF on lipopolysaccharide (LPS)-induced lung NCI-H292 cells, focusing on its role against COVID-19 adsorption. In order to obtain this information, cells treated with LPS, with or without MGF, were analyzed performing wound healing, gene expression of inflammatory cytokines, GSH quantification, and JC-1 staining. Moreover, the inhibition of viral adsorption was evaluated microbiologically and the results were further confirmed by molecular docking analysis. In this regard, MGF downregulates the expression of several inflammatory factors, enhances GSH levels, promotes the wound healing rate, and restores the mitochondrial dysfunction caused by LPS. In addition, MGF significantly inhibits SARS-CoV-2 adsorption as shown by the gene expression of ACE2 and TMPRSS-2, and furtherly confirmed by microbiological and molecular modeling evaluation. Although more investigations are still needed, all data obtained constitute a solid background, demonstrating the cytoprotective role of MGF in inflammatory mechanisms including COVID-19 infection.

## 1. Introduction

The interest towards natural compounds, as an important source for therapeutic approaches, inspired many scientific studies to further investigate the molecular properties of different biomolecular constituents [1,2,3,4,5]. In particular, phytoconstituents seem to be an important source of therapeutic supplements as interesting candidates with anti-inflammatory activity [6,7,8]. Most of the pathological conditions are based on the chronic inflammation of tissues, which represents one of the most common incipits involved in the onset of debilitating diseases including cancer and cardiovascular and pulmonary diseases [6]. Inflammatory diseases include ALI (acute lung injury) or ARDS (acute respiratory distress syndrome), which represents one of the critical pulmonary diseases involving widespread inflammation inducing epithelial injury and lung dysfunction [9,10]. The inflammatory environment and its protraction over time sustain the uncontrolled release of proinflammatory mediators, cytokines including interleukins (ILs), cyclooxygenases (COXs), and metalloproteinases (MMPs), as well as reactive oxygen species (ROS) [11]. The overproduction of ROS is strictly related to increased oxidative stress and cell damage [12]. In terms of lung distress syndrome, severe acute respiratory syndrome coronavirus 2 (SARS-CoV-2) is the agent responsible for COVID-19 disease [13]. Indeed, since the end of 2019, the world has suffered from the onset of the COVID-19 pandemic, which involved a serious global health crisis [14]. The scientific literature widely reported that envelope spike proteins (S) represent the way through which the virus interacts and infects human cells; in fact, the S protein facilitates the binding of viral envelopes to angiotensin-converting enzyme 2 (ACE2) receptors expressed on host cells surfaces [15]. Therefore, many scientific papers suggested the role of natural compounds as strong antioxidant, cytoprotective, and anti-inflammatory agents [16,17], and also as adjuvants of COVID-19 therapy [18]. In addition, functional foods enriched with bioactive molecules may help people overcome this infection by modulating the body’s immune system, generating antiviral activity, and reducing respiratory problems [19]. Among them, mangiferin (2-β-D-glucopyranosyl-1,3,6,7-tetrahydroxy-9H-xanthen-9-one) (MGF) represents an interesting bioactive molecule obtained from mango fruit [20]. It has been widely reported that MGF offers a wide plethora of promising beneficial properties such as antioxidant, antiviral, anti-inflammatory, immunomodulatory, and cytoprotective effects [20,21]. In this study, we aimed to investigate the antioxidant and anti-inflammatory properties of MGF on lipopolysaccharide (LPS)-induced lung mucoepidermoid cells in order to clarify its role in this inflammatory model and its activity in the modulation of ACE2 receptors. In this regard, in silico studies have been carried out to highlight a possible binding between MGF molecules and the spike proteins of SARS-CoV-2.

## 2. Materials and Methods

### 2.1. Cell Culture and Treatments

NCI-H292 [H292] (CRL-1848™) cells were purchased by ATCC and maintained in RPMI-1640 properly supplemented culture medium in growth conditions according to the manufacturer’s data sheet. In order to reproduce the phlogosis in vitro, the cells were incubated in the presence or absence of LPS (*E. coli*; Sigma L431-1MG) at the concentration of 10 μg/mL for 1 h. After the LPS treatment, mangiferin (Sigma M3547-100MG) was added at 20 μg/mL for 24 h. Four groups of treatments were performed as follows: CTRL, LPS 10 μg/mL, MGF 20 μg/mL, and MGF + LPS [22].

### 2.2. Cell Viability

The cells were seeded in 96-multiwell plates and properly treated with different concentrations of MGF (30 μg/mL, 20 μg/mL, 10 μg/mL, 5 μg/mL, and 1 μg/mL). After 24 h of treatment, the medium was replaced by a solution containing bromide 3-(4,5-dimethylthiazol-2-yl)-2,5-diphenyltetrazolium (MTT) salts (Sigma–Merck Life Science, Milan, Italy) and incubated for 3 h at 37 °C. Finally, 100 μL of dimethyl sulfoxide (DMSO) (Sigma–Merck Life Science, Milan, Italy) was added to dissolve formazan crystals and the absorbance was detected at a wavelength of 570 nm [23].

### 2.3. RNA Extraction and Gene Expression

Total RNA extraction was performed using TRIzol (Life Technology, Milan, Italy). The cDNA was synthetized from total mRNA using the Applied Biosystem (Foster City, CA, USA) reverse transcription kit. The quantitative analysis was carried out with the One-Step Fast Real-Time PCR System Applied Biosystem using the SYBR Green PCR master mix (Life Technology, Milan, Italy). The list of primer sequences is shown in Table 1. The relative gene expression levels of each PCR product were obtained according to the threshold cycle (Ct) value and furtherly normalized with the housekeeping gene GAPDH (glyceraldeyde-3-phosphate dehydrogenase) using the comparative 2^−ΔΔCt^ evaluation method [24].

### 2.4. GSH Content Quantification

The measurement of the intracellular content of reduced glutathione (GSH) was obtained using a colorimetric assay based on the reaction of free thiol groups with the reagent 2,2-dithio-bis-nitrobenzoic acid (DTNB) (Sigma–Merck Life Science, Milan, Italy) and subsequent reading at λ = 412 nm (εM = 13,600 M^−1^∙cm^−1^; εM is the molar absorbance coefficient). Every measurement was performed in triplicate using a spectrophotometer (Synergy 2 from BioTek) [25].

### 2.5. Scratch Assay

The repair effect of treatments towards epithelial migration was evaluated by the wound healing assay. Once the cell monolayers were confluent, they were wounded by scratching with a pipette tip and gently washed with PBS 1×; finally, the scratched cells were incubated with the treatment medium (time point 0). The wound closure area was monitored at each time point (t0 h, t6 h, and t24 h) by acquiring photos at 10× magnification using a bright-field microscope from random fields. ImageJ software (Broken Symmetry Software, Bethesda, MD, USA) was used to analyze the wound areas by evaluating the closure of the cellular front over time into the cell-free wound section [26].

### 2.6. Evaluation of Mitochondrial Damage (ΔΨm)

The altered mitochondrial membrane potential (ΔΨm) was detected using the selective lipophilic dye JC-1 (Sigma–Merck Life Science, Milan, Italy). This latter is able to penetrate in mitochondria, producing a reversible fluorescence shift from green to red depending on the mitochondrial membrane polarization. In healthy viable cells with physiological membrane potential, JC-1 is located in the mitochondrial membrane in the form of dimers (red fluorescence), while in the damaged cells with altered membrane potential, the probe remains in the cytoplasm in a monomeric form (green fluorescence). Cells properly treated were incubated with JC-1 in the dark, for 20 min, at a temperature of 37 °C. Finally, the cells were washed in PBS and read spectrophotometrically (625/530 nm) [27].

### 2.7. Inhibition of Viral Adsorption

Green Pseudo SARS-CoV-2 Reporter and Red Fluorescent ACE2 BacMam (Montana Molecular, Bozeman, MT, USA) were used to evaluate the viral adsorption inhibitory capacity of the substances under study. The assay was carried out according to the manufacturer’s protocol, which was optimized for detection on a fluorescence plate reader in adherent A549 cells. Human lung epithelial A549 cells were purchased from the American Tissue Culture Collection (ATCC; Manassas, VA, USA), and cultivated in Dulbecco’s modified Eagle medium (DMEM) supplemented with 10% FBS and drugs (100 U/mL penicillin and 100 μg/mL streptomycin). Briefly, 1.5 × 10^4^ cells/well were plated in their complete media on a 96-well black plate. One row of wells was reserved for control cells that were not transduced with ACE2 BacMam. Then, the plate was incubated under normal growth conditions (5% CO_2_ and 37 °C, protected from light) for 18–24 h. The day after, the transduction mix of ACE2 BacMam in complete media was prepared in order for the cells to express the receptor: 50 μL/well of the transduction mix was added and the plate was incubated again under normal growth conditions for 24–32 h. Subsequently, the media was removed and the plate was washed once with pre-warmed PBS (100 μL/well). Then, 150 µL of transduction mix that included Green Pseudo SARS-CoV-2, fresh complete media, sodium butyrate, and blocking compounds was added to each well, as described in Table 2. The plate was incubated under normal growth conditions (5% CO_2_ and 37 °C, protected from light), for 12–24 h and finally, spectrophotometric readings using BioTek Synergy MX™ (ex/em 488/525 nm) and microscopic analysis with Leica Microsystems DM1000 (20×) were performed to evaluate the activity of the substances analyzed. The evaluation of the inhibitory activity was performed under the following experimental condition: cells exposed simultaneously to Green Pseudo SARS-CoV-2 and substances (LPS, MGF, LPS + MGF). The assay included two internal controls: K cells (cells not exposed to either pseudovirus or substances) and K virus (cells infected with pseudovirus, but not exposed to the substances) as negative and positive internal controls, respectively. 

### 2.8. Molecular Modeling

Using molecular docking techniques, the binding between the MGF and the receptor-binding domain (RBD) of SARS-CoV-2 was shown. The RBD is significant because the amino acid sequence of the spike protein binds to the human ACE2 receptor. This amino acid sequence extends from residue Thr333 to residue Gly526 [28]. To perform docking and molecular dynamics simulations, the crystal structures of SARS-CoV-2 of the spike S1 portion bounded to the ACE2 receptor (PDB code: 6M0J resolution 2.45 Å) were retrieved from the Protein Data Bank. Flare 6.0 software (Cresset^®^, Litlington, Cambridgeshire, UK) was used to edit the proteins for both docking and molecular dynamics studies. The PDB had accessory parts on both the spike protein and ACE receptor removed, leaving only the interface area between the spike protein and the ACE2 receptor. Flare subprogram (protein preparation) was used to prepare the protein for both docking and molecular dynamic studies, applying standard settings. After this step, both portions were relaxed with two cycles of brief (1 × 2 ns) dynamic simulations with a water box created with the same program Flare. The mangiferin (2-β-D-glucopyranosyl-1,3,6,7-tetrahydroxy-9H-xanthan-9-one) structure was built and its energy was minimized with a “Flare preparation ligand”. Docking was conducted using the “Accurate but slow” setting, which applies a higher-precision type of calculation, using more rigorous sampling and scoring algorithms to increase the accuracy and reliability of the predictions. Flare estimates the free energy of protein–ligand binding by providing an additional scoring value called Vscore. Finally, to improve the stability of each complex, short (5ns of production) molecular dynamic runs were performed at a constant temperature, followed by quick minimization of all atoms involved in the binding site. The poses with the best scores and the most plausible ones from the point of view of binding energy were chosen for the molecular dynamics. The molecular dynamic studies were performed using NAMD software (Beckman Institute for Advanced Science and Technology, University of Illinois at Urbana-Champaign, Urbana, IL, USA) in order to perform a short sequence of molecular dynamics calculations. A tLeap water box (TIP3PBOX) was produced. The water protein system (grid dimension: 93 × 70 × 126 Å) was minimized in order to reduce the adaptive issues of the protein to the box during dynamic cycles. During these cycles, 5 ns of NVT and 50 ns of NPT were set and the blocked protein was slowly released into the system. Initially, the protein and the ligand were fixed to achieve good cohesion. After this step, all components were slowly released (backbone and ligand in the first step and then α-carbon) under periodic boundary conditions. This allowed the confirmation of the H-bonds involved and a more accurate investigation of the ligand–receptor complex at the binding site. A completely released system was used (10 cycles for molecule and 100 ns for each cycle) during the production phase.

### 2.9. Statistical Analysis

In order to perform statistical analysis of data, Prism 8.0.2. software (GraphPad Software, San Diego, CA, USA) was selected and the one-way ANOVA test was used to assess significant differences among groups. Statistical significance (*p* < 0.05) of the differences between the experimental groups was determined by the Tukey test for the analysis of multiple comparisons.

## 3. Results

### 3.1. MGF Effect on Cell Viability

**Figure 1:**
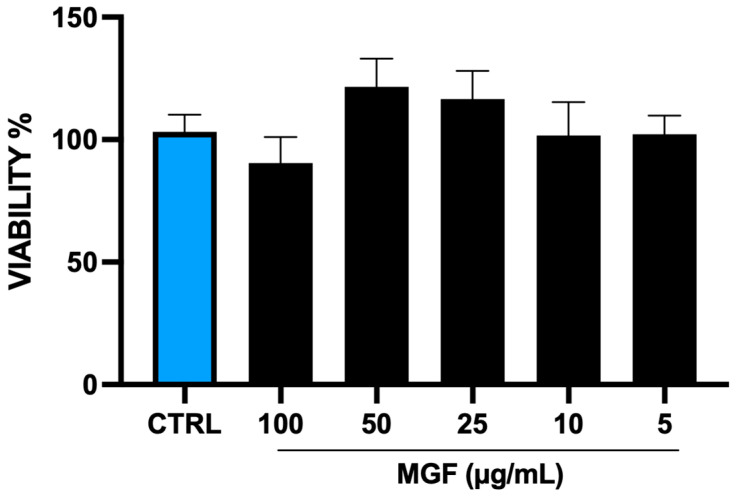
Cell viability determined by MTT assay on NCI-H292 cells, untreated and treated with MGF at different concentrations (30, 20, 10, 5, 1 μg/mL) for 24 h.

In order to assess the eventual toxicity of MGF on H292 cells, an MTT assay was performed (Figure 1). The cells were treated with different concentrations of MGF (from 30 μg/mL to 1 μg/mL). With the only exception of the highest concentration, which resulted in a slight and not significant decrease of cell viability, the other concentrations did not show any toxicity compared to the control. According to the literature, the concentration with the highest viability rate (20 μg/mL) was chosen for the following experiments.

### 3.2. MGF Exhibits Anti-Inflammatory Effects Following LPS Stimulation

**Figure 2:**
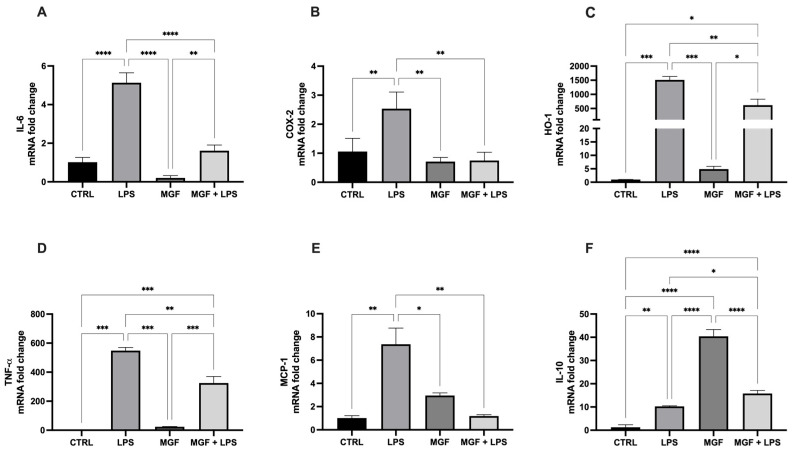
mRNA expression of IL-6 (**A**), COX-2 (**B**), HO-1 (**C**), TNF-α (**D**), MCP-1 (**E**) and IL-10 (**F**) in NCI-H292 cells. Cells were treated with LPS 10 μg/mL for 1 h, and with MGF 20 μg/mL for 24 h. * *p* < 0.05; ** *p* < 0.01; *** *p* < 0.001; **** *p* < 0.0001.

As shown in Figure 2, the exposure to LPS was able to induce the gene expression of the markers closely related to inflammation, such as interleukin 6 (IL-6), prostaglandin-endoperoxide synthase (COX-2), heme oxygenase 1 (HO-1), tumor necrosis factor-α (TNF-α) and monocyte chemoattractant protein-1 (MCP-1). Due to the post-treatment with MGF, the expression of such genes was significantly reduced. In addition, the MGF treatment alone strongly induced interleukin 10 (IL-10) levels, involved in anti-inflammatory processes (Figure 2F); the MGF administration following LPS pre-treatment was able to cause a slight, but significant, increase in the IL-10 level compared to LPS alone.

### 3.3. Activity of MGF on Host Cell Entry System Used by SARS-CoV-2

**Figure 3:**
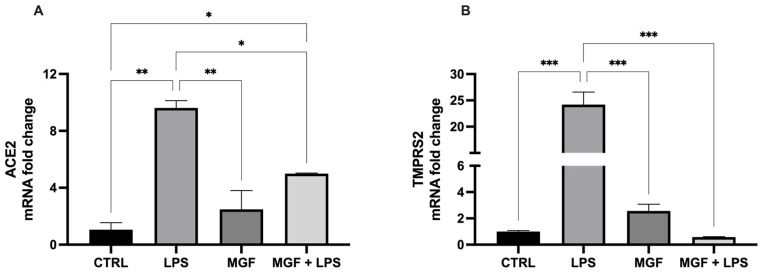
mRNA expression of ACE2 (**A**) and TMPRSS2 (**B**) in NCI-H292 cells. Cells were treated with LPS 10 μg/mL for 1 h, and with MGF 20 μg/mL for 24 h. * *p* < 0.05; ** *p* < 0.01; *** *p* < 0.001.

In order to evaluate the efficacy of MGF on the expression of the main factors that contribute to the virulence of SARS-CoV-2 and pathogenesis of coronavirus disease-19 (COVID-19), angiotensin-converting enzyme 2 (ACE2), and trans-membrane protease serine 2 (TMPRSS2), changes in their mRNA levels were measured following the treatments (Figure 3). The data showed that LPS was able to markedly induce both ACE2 and TMPRSS2 levels, whereas the combination with MGF significantly reduced their expression: in particular, the ACE2 levels were halved, while TMPRSS2 expression was decreased to the control level.

### 3.4. MGF Maintains Redox Balance by Increasing GSH Levels

**Figure 4:**
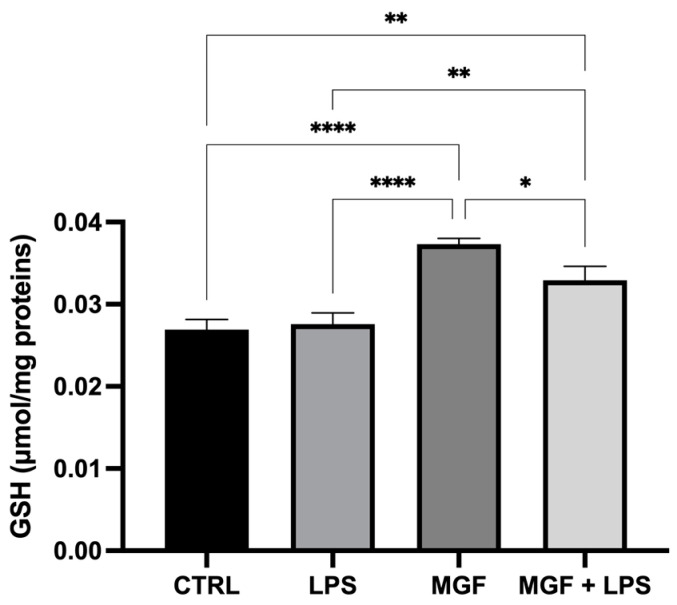
Quantification of intracellular content of reduced GSH analyzed by spectrophotometric assay in NCI-H292 cells. Cells were treated with LPS 10 μg/mL for 1 h, and with MGF 20 μg/mL for 24 h. * *p* < 0.05; ** *p* < 0.01; **** *p* < 0.0001.

The results reported in Figure 4 show that MGF alone was able to increase GSH basal levels compared to the control. The co-treatment with MGF and LPS resulted in a minor enhancement of GSH levels, still regulating the oxidative stress occurring during the inflammatory condition, although the LPS single treatment did not affect the redox balance of the cells.

### 3.5. MGF Increases Wound Healing following LPS Stimulation

**Figure 5:**
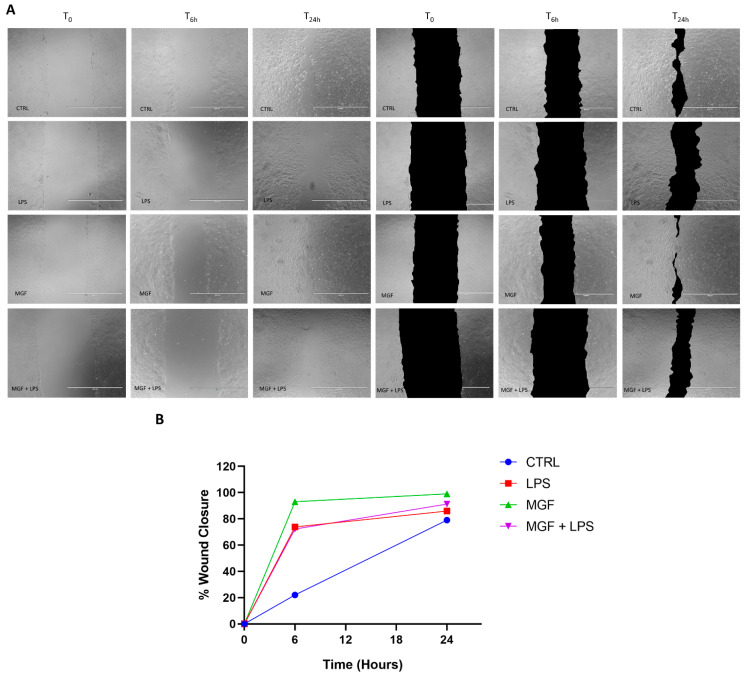
Promoting effects of MGF on wound repairing in presence of LPS in NCI-H292 cells. (**A**) representative images of wound healing assays (**left**) with highlight of wound closing area (**right**). Cells were treated with LPS 10 μg/mL and with MGF 20 μg/mL immediately after the scratch at 0 h, 6 h, and 24 h of incubation. (**B**) Relative quantification of wound closure. Scale bar = 1000 µm.

A standard wound healing test was used in order to study the impact of our treatments on wound repair capability. As shown in Figure 5, Panels A and B, the MGF treatment produced a significant increase in the wound closure rate compared to the control, from about 20% (CTRL) to about 90% at 6 h, and from about 75% (CTRL) to about 100% at 24 h. According to the literature, the LPS treatment enhanced the wound healing rate as well, despite not reaching the MGF rate (about 70% at 6 h and about 85% at 24 h). Finally, the MGF administration after the pre-treatment with LPS ameliorated the repairing rate compared to LPS alone, reaching about 90% at 24 h, despite not providing differences at 6 h.

### 3.6. MGF Restores LPS Mitochondrial Dysfunction

**Figure 6:**
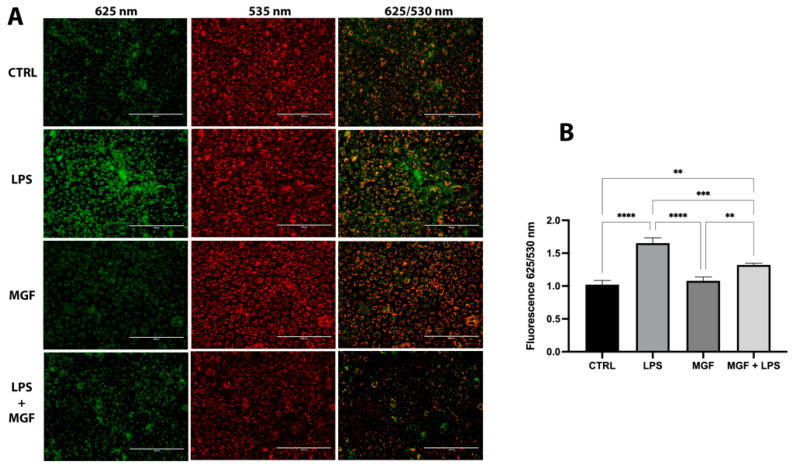
(**A**) Fluorescence images of JC-1 staining after 6 h of treatment; scale bar = 200 μm. (**B**) Effect of MGF and LPS on mitochondrial membrane potential. Cells were treated with LPS 10 μg/mL for 1 h, and MGF 20 μg/mL for 5 h. ** *p* < 0.01; *** *p* < 0.001; **** *p* < 0.0001.

The higher presence of JC-1 monomer (green fluorescence) in LPS-treated cells, compared to CTRL and MGF groups, indicated mitochondrial dysfunction due to alterations in the mitochondrial membrane potential. As shown in Figure 6, the co-treatment of MGF and LPS was able to reduce the ratio of the red/green fluorescence signal, highlighting a protective effect of MGF against LPS-induced mitochondrial stress.

### 3.7. MGF Activity on SARS-CoV-2 Adsorption

**Figure 7:**
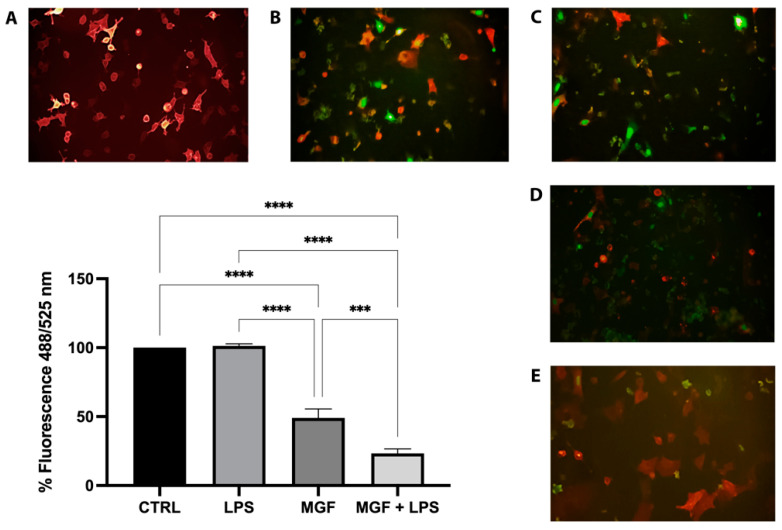
Representative images of pseudo SARS-CoV-2 entry: entry of pseudovirus bearing the green reporter in red fluorescence ACE2-expressing host cells A549. Green fluorescence nucleus was only expressed in cells infected by the pseudovirus; therefore, the amount of green present was proportional to the number of infected cells. (**A**) K cells, A549 transduced with ACE2 BacMam; (**B**) K virus, cells infected by pseudo SARS-CoV-2; (**C**) LPS treated cells; (**D**) MGF treated cells; (**E**) co-treated MGF + LPS cells. Images obtained at 20X magnification. *** *p* < 0.001; **** *p* < 0.0001.

The spike protein on the surface of SARS-CoV-2 interacts with the ACE2 protein expressed on the surface of human cells to mediate viral entry into the host cell. Indeed, the tight binding of the spike protein to ACE2 is basic for the infection process. In this context, the first step was to enable the A549 cells to express ACE2. The transduction of A549 cells treated with ACE2 BacMam was confirmed by the fluorescence emitted after 24 h (Figure 7A). Furthermore, pseudovirus affinity for ACE2 was also confirmed (Figure 7B). Therefore, we submitted LPS (10 µg/mL) and MGF (20 µg/mL) to the inhibition of a viral adsorption assay in order to determine the inhibition of spike-ACE2 protein–protein interaction. The high presence of pseudovirus SARS-CoV-2 in the LPS-treated cell nuclei indicated viral entry equal to the K virus (Figure 7C). Contrariwise, MGF treatment showed a moderate adsorption inhibition (Figure 7D). Moreover, the co-action of the two molecules was tested, and as shown in Figure 7E, the co-treatment of MGF + LPS showed a significant inhibitory activity. Fluorescence quantification was achieved by spectrophotometric reading with BioTek Synergy MX™. The analysis revealed a decrease in fluorescence equal to 0% and 51% for LPS 10 µg/mL and MGF 20 µg/mL, respectively. Furthermore, LPS + MGF showed a 77% decrease compared to the K virus.

### 3.8. Molecular Modeling Studies of MGF on SARS-CoV-2

**Figure 8:**
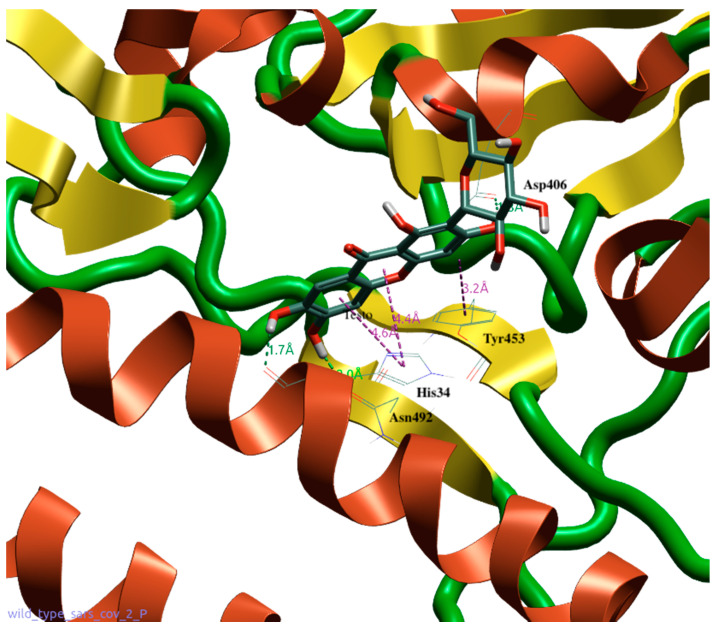
Docking position of MGF (1) in the binding interface between spike 1 (green and yellow) and ACE2 (red). Interaction with His34, Arg457, and His458. Water and accessory parts of spike and ACE2 were omitted for clarity.

**Figure 9:**
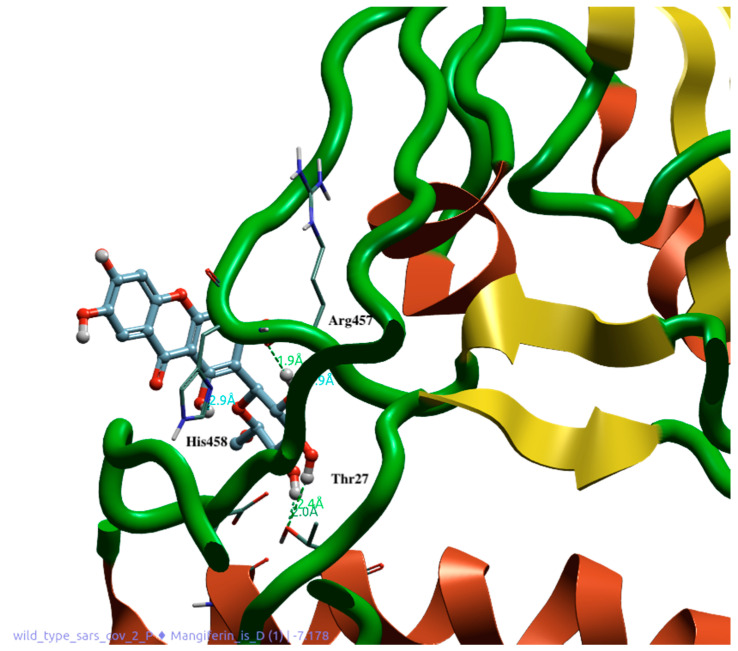
Docking position of MGF (2) at the accessory site between spike 1 (green and yellow) and ACE2 (red). Interactions with Thr27, Asn492, Tyr453, and Asp406. Water and accessory parts of spike and ACE2 were omitted for clarity.

Molecular docking and dynamics studies provided very interesting results. In fact, they show how a naturally derived polyphenol can interfere with the binding between the SARS-CoV-2 spike protein and ACE2 receptor, thereby preventing the entry of the virus into the cells. MGF shows excellent dG (−8948) and VSscore (−10,115), and its structure, which contains several phenolic groups, allows the establishment of several bonds with the amino acids of the spike protein. The hydroxylic groups in MGF form very stable hydrogen bonds with *Asp406* and *Asn492* (Figure 8). The molecule also maintains its position through the Ar–Ar bond between the compound rings and the phenolic portion of *Tyr453*. The molecule also establishes hydrogen and Ar–Ar bonds with *His34* of the ACE2 protein. The presence of a second accessory pocket was revealed using the Flare software during the pre-docking analysis of the complex. This pocket appears to be important for the stability of the anchorage of the spike on the ACE2 surface because a number of bonds were revealed between these two; therefore, we decided to evaluate the action of mangiferin on this pocket. The glycosidic portion of the second molecule throughout the dynamics establishes H-bonds with *Arg457* and *His458* of the spike protein and *Glu23* and *Thr27* of the ACE2 protein, leaving the aromatic portion and hydroxyl groups at the interface with water (Figure 9). 

The dynamics studies showed the capacity of two molecules of MGF to interact with two different anchoring pockets between spike and ACE2. In particular, for the first molecule of MGF at the first binding site, it was observed that the bonding between the amino acids at the interface and the molecule was maintained for the duration of the dynamics. This was possible because of the maintenance of hydrogen bonds between the hydroxyl groups of the rings and the aforementioned amino acids. The glucosyl residue is water exposed for all 100 ns of the dynamic simulation. The second molecule of MGF is inserted into this accessory pocket, showing the same behavior of the first one with the formed bonds maintained for the duration of the dynamic. 

**Figure 10:**
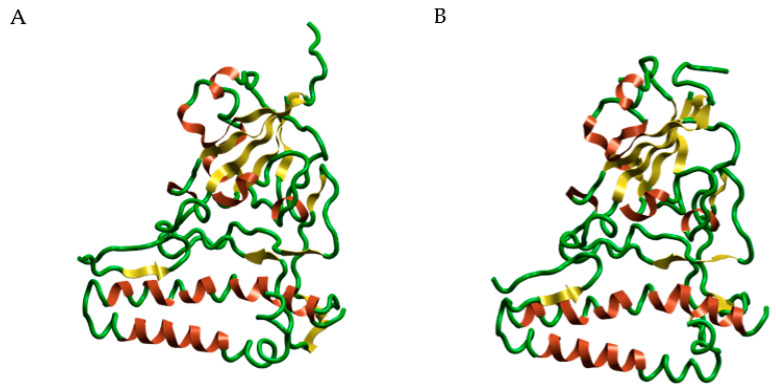
Dynamics studies of MGF (1) and MGF (2) with synergic activity. Panel (**A**) shows the normal structure of the amino acid from Gln388 to Ala443. Panel (**B**) shows the structure of the same portion after the disrupting activity of the synergism of the two MGF molecules. Water and accessory parts of spike and ACE2 were omitted for clarity.

The dynamics studies carried out with two MGF molecules showed a change in the conformation of the second molecule, which, around the 20th ns, changes its conformation by exposing the glucosidic residue to water. Correspondingly, probably due to the synergistic effect of the two MGF molecules, a conformational change that involves amino acids from *Gln*388 to *Ala*443 is shown. This movement of amino acids was evidenced around the 20th ns of dynamic simulation (Figure 10).

## 4. Discussion

The role of natural products and their importance as supplements in many therapeutic approaches represent one of the most popular scientific research fields [23,29,30,31,32,33]. Indeed, the use of natural compounds has been recommended in the recent health emergency of the COVID-19 pandemic [18]. The agent responsible for COVID-19 is severe acute respiratory syndrome coronavirus 2 (SARS-CoV-2), which promotes lung distress syndrome due to a serious chronic inflammatory pattern [13]. The protraction of the inflammatory environment over time sustains the uncontrolled release of proinflammatory mediators (cytokine storm) [34], including ILs, COXs, and MMPs, as well as ROS [11]. The ROS overproduction is strictly related to increased oxidative stress, cell damage [12], and mitochondrial dysfunction [35]. In this context, MGF appears to be a powerful bioactive molecule whose activity is involved in a diversified range of therapeutical applications [36]. In particular, many pharmacological studies demonstrate numerous MGF activities including immunomodulatory, antimicrobial, antiviral, and anti-inflammatory properties [36,37,38]. In this study, we aimed to investigate the antioxidant and anti-inflammatory effects of MGF towards LPS-induced inflammation in human lung cells. Moreover, based on this evidence, we further analyzed MGF’s role against SARS-CoV-2 adsorption both from the microbiological and mechanistic point of view. In particular, during the first step of our study, we performed our investigations using an LPS-induced stress in vitro model on the human lung cell line NCI-H292. Indeed, it is already well established that LPS, the bacterial endotoxin par excellence, triggers massive inflammatory responses caused mainly by mitochondrial abnormalities [39]. The preliminary analysis focused on the evaluation of the cell viability using different concentrations of MGF, in order to verify the absence of cytotoxicity. As shown in Figure 1, MGF does not show cytotoxicity in our cell line and according to the literature, the concentration with the highest viability rate (20 μg/mL) was chosen for the following experiments. Moreover, the scientific literature well recognizes MGF efficacy in terms of anti-inflammatory and antioxidant efficacy. In this regard, in vivo studies demonstrated that MGF, administrated in rats, enhanced both antioxidant enzyme levels and inhibited NF-κB p65 activation, showing a significant reduction of inflammation and cell proliferation [40,41]. Similarly, the same evidence was supported by data obtained from in vitro studies, suggesting that MGF treatment produced in decrease in proinflammatory factors and ROS promoted by LPS stress in different macrophage cell lines [42,43]. In this regard, our data confirm significant anti-inflammatory activity, demonstrated by the different investigations performed on the gene expression of several proinflammatory and anti-inflammatory cytokines. In particular, MGF restored the levels of proinflammatory cytokines including IL6 (Figure 2A), COX2 (Figure 2B), TNFα (Figure 2D), and MCP-1 (Figure 2E), both alone and in combination with LPS compared to the single LPS treatment. Likewise, MGF upregulated the expression of the anti-inflammatory cytokine IL-10 (Figure 2F) and modulated the overexpression of the enzyme HO-1 due to the LPS-induced stress (Figure 2C) [44,45,46,47]. Furthermore, the evaluation of the antioxidant activity was confirmed by GSH (reduced glutathione) quantification, a critical parameter in the analysis of oxidative stress, which actively participates, as a radical scavenger, in the detoxification of the cell environment [48]. The data obtained (Figure 4) showed that MGF is strictly involved in antioxidant cell defense, demonstrating a significant increase in intracellular GSH content compared to LPS treatment. In order to further evaluate the cytoprotective and proliferative effect of MGF against LPS-induced damage, we subsequently performed a wound healing assay, through which it was possible to obtain a clear biological perspective in terms of the cell proliferation rate. In particular, as shown in Figure 5, MGF, both alone and combined with LPS, was demonstrated to significantly enhance the cell proliferation rate compared to LPS treatment, which effectively resulted in a reduction in the wound closure rate compared to the control. Indeed, the restorative activity and the promotion of cell proliferation exerted by MGF are widely reported by some studies, focusing on the regenerative attitude of this xanthonoid mainly related to the involvement of cell cycle factors [49,50]. The numerous beneficial and cytoprotective evidence offered by MGF led us to further investigate its potential involvement in eventual mitochondrial dynamics. Mitochondria seem to play a pivotal role in the immune response due to cell damage input, behaving like a sort of control unit of inflammatory response in case of infection. In fact, pathogenic microorganisms such as viruses are usually detected by specific cell protein complexes through the recognition of pathogen-associated molecular patterns (PAMPs), including LPS [51]. In this context, mitochondria are involved both in the recognition of pathogens and in many mechanisms of inflammatory response, inducing the subsequent recruitment of several proinflammatory cytokines [52]. Specifically, the mitochondrial membrane potential (MMP) results increased following the pro-inflammatory effect of LPS [53]. Indeed, our JC-1 data perfectly supported the scientific literature, since LPS treatment showed the enhancement of MMP as clearly reported in Figure 6. In addition, MGF combined with LPS produced the restoration of MMP to lower levels, comparable to the control. Our in vitro results encouraged us to further investigate the effects of MGF towards COVID-19 infection, focusing on the potential involvement of the virus adsorption mechanism in the host cell. To go into detail, we used BacMam to evaluate the inhibition of SARS-CoV-2 adsorption. BacMam is a modified baculovirus produced using Sf9 insect cells and it is pseudotyped to infect mammalian cells [54,55,56]. The pseudo SARS-CoV-2 enters host cells through interactions with ACE2, but once inside the cell, it does not replicate or integrate into the host cell genome. This enables the screening of blocking agents to be performed in a few days and without the appropriate guidelines for handling and processing samples associated with SARS-CoV-2 [57]. The anti-SARS-CoV-2 action of MGF was demonstrated in Figure 7. The green fluorescence in the nuclei of the host cells indicated the entry of pseudoviruses, while the viral entry block showed an evident decrease in fluorescence. In support of these results, MGF treatment demonstrated a significant downregulation of ACE2 and TMPRRS2 genes compared to LPS upregulation, further confirming MGF’s anti-infective potential in vitro (Figure 3). The data obtained show a significant ability of MGF to prevent the virus entrance both in the absence or presence of LPS, as shown in Figure 7 (panels D and E, respectively), demonstrating an enhanced antiviral efficacy of MGF. In order to further investigate the potential role of MGF, anti-SARS-CoV-2 activity has been also demonstrated in in silico studies performing molecular docking analysis. 

The MGF binding to amino acids of the RBD of the spike protein allows its interposition at the ACE2 receptor binding site, potentially inhibiting the viral attachment and entry into the cells. Molecular dynamics studies indicated that the binding of the MGF persisted for at least 100 ns from the onset of contact. This implies that the established bond is well consolidated, as shown by the number and type of amino acid bonds. In addition, the analysis carried out in the presence of two molecules of MGF showed that binding to the second accessory pocket caused disruption of the spike protein and movement of several amino acids (Figure 8 and Figure 9). The disrupting activity of the synergism due to the two MGF molecules is clearly reported by Figure 10. Dose-dependent use might explain this behavior of MGF to also bind to an accessory site and disrupt S1. Although further studies are needed to investigate more biological fields and to understand the behavior of MGF to prevent S1 binding to ACE2, preventing virus entry into cells, our results constitute a solid starting point, confirming the protective role of MGF in inflammatory mechanisms, including COVID-19 infection. 

## 5. Conclusions

The growing interest in natural compounds motivated us to investigate the potential role of MGF in a model of lung inflammation induced by LPS, focusing specifically on SARS-CoV-2 adsorption mechanisms. MGF’s beneficial effects have been widely confirmed by the data obtained. Interestingly, MGF was shown to exert cytoprotective, antioxidant, and anti-inflammatory activities, as evidenced by the decreased gene expression of inflammatory markers, such as IL-6, COX-2, and TNF-α, and increased intracellular reduced GSH. The significant inhibitory efficacy of MGF against SARS-CoV-2 adsorption in host cells was also confirmed by the results shown in vitro in A549 cells and confirmed by the docking and molecular dynamics results, which show the positioning of the molecule at the interface between ACE2 and the spike protein. This positioning of the MGF molecule could explain its inhibitory efficacy against the adsorption of SARS-CoV-2. Although more investigations are needed to obtain more biological details and to further confirm the evidence provided so far, we can consider MGF an interesting bioactive molecule whose properties make it a potential therapeutic adjuvant in pathological phlogistic contexts such as COVID-19 infection.

## Figures and Tables

**Table 1:** Primer sequences used to assess gene expression in human cells.

Gene	Forward	Reverse
IL-6	CCACCGGGAACGAAAGAGAA	GAGAAGGCAACTGGACCGAA
IL-10	CCACAAGACAGACTTGCAAAAG	AACAAGTTGTCCAGCTGATCC
COX-2	CTGGCGCTCAGCCATACAG	CGCACTTATACTGGTCAAATCCC
HO-1	GTTGGGGTGGTTTTTGAGCC	TTAGACCAAGGCCACAGTGC
TNF-α	GCAACAAGACCACCACTTCG	GATCAAAGCTGTAGGCCCCA
MCP-1	CCTTCATTCCCCAAGGGCTC	GGTTTGCTTGTCCAGGTGGT
ACE2	TGGAGAAAATCCTTATGCCTCCA	CTCTCCTTGGCCATGTTGTCT
TMPRSS2	GTAGGACCAGCCTCCATTTCC	CCTCACCTTTGGTCCTCTGAC
GAPDH	TTCTTTTGCGTCGCCAGCC	CTTCCCGTTCTCAGCCTTGAC

**Table 2:** Pseudo SARS-CoV-2 transduction mix.

	Amount per Well	Final Concentration
Pseudovirus SARS-CoV-2	2.5 μL	3.3 × 108 VG/mL
Sodium butyrate	0.6 μL	2 mM
Compounds with complete media	Adjust to 150 μL	10 µg/mL LPS (LPS); 20 µg/mL mangiferin (MGF); LPS + MGF

## Abbreviations

ACE2	Angiotensin-converting enzyme 2
ALI	Acute lung injury
ARDS	Acute respiratory distress syndrome
COX-2	Prostaglandin-endoperoxide synthase
COXs	Cyclooxygenases
CTRL	Control
DMSO	Dimethyl sulfoxide
DTNB	2,2-dithio-bis-nitrobenzoic acid
GSH	Reduced glutathione
HO-1	Heme oxygenase 1
IL-10	Interleukin 10
IL-6	Interleukin 6
ILs	Interleukins
LPS	Lipopolysaccharide
MCP-1	Monocyte chemoattractant protein-1
MGF	Mangiferin
MMPs	Metalloproteinases
MTT	Bromide 3-(4,5-dimethylthiazol-2-yl)-2,5-diphenyltetrazolium
RBD	Receptor binding domain
ROS	Reactive oxygen species
SARS-CoV-2	Severe acute respiratory syndrome coronavirus 2
TMPRSS2	Trans-membrane protease serine 2
TNF-α	Tumor necrosis factor-alpha

## References

[1] Kim M.H., Lee S.M., An K.W., Lee M.J., Park D.H. (2021). Usage of Natural Volatile Organic Compounds as Biological Modulators of Disease. Int. J. Mol. Sci..

[2] Rodrigues T., Reker D., Schneider P., Schneider G. (2016). Counting on natural products for drug design. Nat. Chem..

[3] Miceli M., Bontempo P., Nebbioso A., Altucci L. (2014). Natural compounds in epigenetics: A current view. Food Chem. Toxicol..

[4] Barbagallo I., Vanella L., Cambria M.T., Tibullo D., Godos J., Guarnaccia L., Zappala A., Galvano F., Li Volti G. (2015). Silibinin Regulates Lipid Metabolism and Differentiation in Functional Human Adipocytes. Front. Pharmacol..

[5] Palmeri R., Monteleone J.I., Spagna G., Restuccia C., Raffaele M., Vanella L., Li Volti G., Barbagallo I. (2016). Olive Leaf Extract from Sicilian Cultivar Reduced Lipid Accumulation by Inducing Thermogenic Pathway during Adipogenesis. Front. Pharmacol..

[6] Patil K.R., Mahajan U.B., Unger B.S., Goyal S.N., Belemkar S., Surana S.J., Ojha S., Patil C.R. (2019). Animal Models of Inflammation for Screening of Anti-inflammatory Drugs: Implications for the Discovery and Development of Phytopharmaceuticals. Int. J. Mol. Sci..

[7] Li Volti G., Musumeci T., Pignatello R., Murabito P., Barbagallo I., Carbone C., Gullo A., Puglisi G. (2012). Antioxidant potential of different melatonin-loaded nanomedicines in an experimental model of sepsis. Exp. Biol. Med..

[8] Vanella L., Barbagallo I., Acquaviva R., Di Giacomo C., Cardile V., Abraham N.G., Sorrenti V. (2013). Ellagic acid: Cytodifferentiating and antiproliferative effects in human prostatic cancer cell lines. Curr. Pharm. Des..

[9] Matthay M.A., Zemans R.L. (2011). The acute respiratory distress syndrome: Pathogenesis and treatment. Annu. Rev. Pathol..

[10] Huppert L.A., Matthay M.A., Ware L.B. (2019). Pathogenesis of Acute Respiratory Distress Syndrome. Semin. Respir. Crit. Care Med..

[11] Kellner M., Noonepalle S., Lu Q., Srivastava A., Zemskov E., Black S.M. (2017). ROS Signaling in the Pathogenesis of Acute Lung Injury (ALI) and Acute Respiratory Distress Syndrome (ARDS). Adv. Exp. Med. Biol..

[12] Rahman I., Adcock I.M. (2006). Oxidative stress and redox regulation of lung inflammation in COPD. Eur. Respir. J..

[13] Lima-Martinez M.M., Carrera Boada C., Madera-Silva M.D., Marin W., Contreras M. (2021). COVID-19 and diabetes: A bidirectional relationship. Clin. Investig. Arterioscler..

[14] Majumder J., Minko T. (2021). Recent Developments on Therapeutic and Diagnostic Approaches for COVID-19. AAPS J..

[15] Mohamadian M., Chiti H., Shoghli A., Biglari S., Parsamanesh N., Esmaeilzadeh A. (2021). COVID-19: Virology, biology and novel laboratory diagnosis. J. Gene Med..

[16] Wu X.X., Huang X.L., Chen R.R., Li T., Ye H.J., Xie W., Huang Z.M., Cao G.Z. (2019). Paeoniflorin Prevents Intestinal Barrier Disruption and Inhibits Lipopolysaccharide (LPS)-Induced Inflammation in Caco-2 Cell Monolayers. Inflammation.

[17] Sul O.J., Ra S.W. (2021). Quercetin Prevents LPS-Induced Oxidative Stress and Inflammation by Modulating NOX2/ROS/NF-kB in Lung Epithelial Cells. Molecules.

[18] Parisi G.F., Carota G., Castruccio Castracani C., Spampinato M., Manti S., Papale M., Di Rosa M., Barbagallo I., Leonardi S. (2021). Nutraceuticals in the Prevention of Viral Infections, including COVID-19, among the Pediatric Population: A Review of the Literature. Int. J. Mol. Sci..

[19] Goli M. (2020). Review of novel human beta-coronavirus (2019-nCoV or SARS-CoV-2) from the food industry perspective-Appropriate approaches to food production technology. Food Sci. Nutr..

[20] Imran M., Arshad M.S., Butt M.S., Kwon J.H., Arshad M.U., Sultan M.T. (2017). Mangiferin: A natural miracle bioactive compound against lifestyle related disorders. Lipids Health Dis..

[21] Dar A., Faizi S., Naqvi S., Roome T., Zikr-ur-Rehman S., Ali M., Firdous S., Moin S.T. (2005). Analgesic and antioxidant activity of mangiferin and its derivatives: The structure activity relationship. Biol. Pharm. Bull..

[22] Di Stefano A., Dossena F., Gnemmi I., D’Anna S.E., Brun P., Balbi B., Piraino A., Spanevello A., Nucera F., Carriero V. (2022). Decreased humoral immune response in the bronchi of rapid decliners with chronic obstructive pulmonary disease. Respir. Res..

[23] Sferrazzo G., Palmeri R., Restuccia C., Parafati L., Siracusa L., Spampinato M., Carota G., Distefano A., Di Rosa M., Tomasello B. (2022). *Mangifera indica* L. Leaves as a Potential Food Source of Phenolic Compounds with Biological Activity. Antioxidants.

[24] Raffaele M., Barbagallo I., Licari M., Carota G., Sferrazzo G., Spampinato M., Sorrenti V., Vanella L. (2018). N-Acetylcysteine (NAC) Ameliorates Lipid-Related Metabolic Dysfunction in Bone Marrow Stromal Cells-Derived Adipocytes. Evid. Based Complement. Altern. Med..

[25] Spampinato M., Sferrazzo G., Pittala V., Di Rosa M., Vanella L., Salerno L., Sorrenti V., Carota G., Parrinello N., Raffaele M. (2020). Non-competitive heme oxygenase-1 activity inhibitor reduces non-small cell lung cancer glutathione content and regulates cell proliferation. Mol. Biol. Rep..

[26] D’Angeli F., Guadagni F., Genovese C., Nicolosi D., Trovato Salinaro A., Spampinato M., Mannino G., Lo Furno D., Petronio Petronio G., Ronsisvalle S. (2021). Anti-Candidal Activity of the Parasitic Plant Orobanche crenata Forssk. Antibiotics.

[27] Perelman A., Wachtel C., Cohen M., Haupt S., Shapiro H., Tzur A. (2012). JC-1: Alternative excitation wavelengths facilitate mitochondrial membrane potential cytometry. Cell Death Dis..

[28] Lan J., Ge J., Yu J., Shan S., Zhou H., Fan S., Zhang Q., Shi X., Wang Q., Zhang L. (2020). Structure of the SARS-CoV-2 spike receptor-binding domain bound to the ACE2 receptor. Nature.

[29] Ronsisvalle S., Lissandrello E., Fuochi V., Petronio Petronio G., Straquadanio C., Crasci L., Panico A., Milito M., Cova A.M., Tempera G. (2019). Antioxidant and antimicrobial properties of Casteanea sativa Miller chestnut honey produced on Mount Etna (Sicily). Nat. Prod. Res..

[30] Barbagallo I., Vanella L., Distefano A., Nicolosi D., Maravigna A., Lazzarino G., Di Rosa M., Tibullo D., Acquaviva R., Li Volti G. (2016). Moringa oleifera Lam. improves lipid metabolism during adipogenic differentiation of human stem cells. Eur. Rev. Med. Pharmacol. Sci..

[31] Fuochi V., Barbagallo I., Distefano A., Puglisi F., Palmeri R., Di Rosa M., Giallongo C., Longhitano L., Fontana P., Sferrazzo G. (2019). Biological properties of Cakile maritima Scop. (Brassicaceae) extracts. Eur. Rev. Med. Pharmacol. Sci..

[32] Vanella L., Tibullo D., Godos J., Pluchinotta F.R., Di Giacomo C., Sorrenti V., Acquaviva R., Russo A., Li Volti G., Barbagallo I. (2016). Caffeic Acid Phenethyl Ester Regulates PPAR’s Levels in Stem Cells-Derived Adipocytes. PPAR Res..

[33] Presti S., Manti S., Parisi G.F., Papale M., Barbagallo I.A., Li Volti G., Leonardi S. (2021). Lactoferrin: Cytokine Modulation and Application in Clinical Practice. J. Clin. Med..

[34] Muhammad M., Hassan T.M., Baba S.S., Radda M.I., Mutawakkil M.M., Musa M.A., AbuBakar S., Loong S.K., Yusuf I. (2022). Exploring NFkappaB pathway as a potent strategy to mitigate COVID-19 severe morbidity and mortality. J. Public Health Afr..

[35] Zorov D.B., Juhaszova M., Sollott S.J. (2014). Mitochondrial reactive oxygen species (ROS) and ROS-induced ROS release. Physiol. Rev..

[36] Matkowski A., Kus P., Goralska E., Wozniak D. (2013). Mangiferin—A bioactive xanthonoid, not only from mango and not just antioxidant. Mini Rev. Med. Chem..

[37] Vyas A., Syeda K., Ahmad A., Padhye S., Sarkar F.H. (2012). Perspectives on medicinal properties of mangiferin. Mini Rev. Med. Chem..

[38] Nunez Selles A.J., Daglia M., Rastrelli L. (2016). The potential role of mangiferin in cancer treatment through its immunomodulatory, anti-angiogenic, apoptopic, and gene regulatory effects. Biofactors.

[39] Kielian T.L., Blecha F. (1995). CD14 and other recognition molecules for lipopolysaccharide: A review. Immunopharmacology.

[40] Wang X., Yuwen T., Yanqin T. (2021). Mangiferin Inhibits Inflammation and Cell Proliferation, and Activates Proapoptotic Events via NF-kappaB Inhibition in DMBA-Induced Mammary Carcinogenesis in Rats. J. Environ. Pathol. Toxicol. Oncol..

[41] Dong M., Li L., Li G., Song J., Liu B., Liu X., Wang M. (2020). Mangiferin protects against alcoholic liver injury via suppression of inflammation-induced adipose hyperlipolysis. Food Funct..

[42] Lei L.Y., Wang R.C., Pan Y.L., Yue Z.G., Zhou R., Xie P., Tang Z.S. (2021). Mangiferin inhibited neuroinflammation through regulating microglial polarization and suppressing NF-kappaB, NLRP3 pathway. Chin. J. Nat. Med..

[43] Bulugonda R.K., Kumar K.A., Gangappa D., Beeda H., Philip G.H., Muralidhara Rao D., Faisal S.M. (2017). Mangiferin from Pueraria tuberosa reduces inflammation via inactivation of NLRP3 inflammasome. Sci. Rep..

[44] Barbagallo I., Marrazzo G., Frigiola A., Zappala A., Li Volti G. (2012). Role of carbon monoxide in vascular diseases. Curr. Pharm. Biotechnol..

[45] Sorrenti V., Pittala V., Romeo G., Amata E., Dichiara M., Marrazzo A., Turnaturi R., Prezzavento O., Barbagallo I., Vanella L. (2018). Targeting heme Oxygenase-1 with hybrid compounds to overcome Imatinib resistance in chronic myeloid leukemia cell lines. Eur. J. Med. Chem..

[46] Li Volti G., Tibullo D., Vanella L., Giallongo C., Di Raimondo F., Forte S., Di Rosa M., Signorelli S.S., Barbagallo I. (2017). The Heme Oxygenase System in Hematological Malignancies. Antioxid. Redox Signal..

[47] Barbagallo I., Tibullo D., Di Rosa M., Giallongo C., Palumbo G.A., Raciti G., Campisi A., Vanella A., Green C.J., Motterlini R. (2008). A cytoprotective role for the heme oxygenase-1/CO pathway during neural differentiation of human mesenchymal stem cells. J. Neurosci. Res..

[48] Owen J.B., Butterfield D.A. (2010). Measurement of oxidized/reduced glutathione ratio. Methods Mol. Biol..

[49] Wang H.L., Li C.Y., Zhang B., Liu Y.D., Lu B.M., Shi Z., An N., Zhao L.K., Zhang J.J., Bao J.K. (2014). Mangiferin facilitates islet regeneration and beta-cell proliferation through upregulation of cell cycle and beta-cell regeneration regulators. Int. J. Mol. Sci..

[50] Sekiguchi Y., Mano H., Nakatani S., Shimizu J., Kataoka A., Ogura K., Kimira Y., Ebata M., Wada M. (2017). Mangiferin positively regulates osteoblast differentiation and suppresses osteoclast differentiation. Mol. Med. Rep..

[51] Andrieux P., Chevillard C., Cunha-Neto E., Nunes J.P.S. (2021). Mitochondria as a Cellular Hub in Infection and Inflammation. Int. J. Mol. Sci..

[52] Dinarello C.A. (2011). Interleukin-1 in the pathogenesis and treatment of inflammatory diseases. Blood.

[53] Mills E.L., Kelly B., Logan A., Costa A.S.H., Varma M., Bryant C.E., Tourlomousis P., Dabritz J.H.M., Gottlieb E., Latorre I. (2016). Succinate Dehydrogenase Supports Metabolic Repurposing of Mitochondria to Drive Inflammatory Macrophages. Cell.

[54] Azali M.A., Mohamed S., Harun A., Hussain F.A., Shamsuddin S., Johan M.F. (2022). Application of Baculovirus Expression Vector system (BEV) for COVID-19 diagnostics and therapeutics: A review. J. Genet. Eng. Biotechnol..

[55] Chalfie M., Tu Y., Euskirchen G., Ward W.W., Prasher D.C. (1994). Green fluorescent protein as a marker for gene expression. Science.

[56] Kost T.A., Condreay J.P., Ames R.S., Rees S., Romanos M.A. (2007). Implementation of BacMam virus gene delivery technology in a drug discovery setting. Drug Discov. Today.

[57] Centers for Disease Control and Prevention, NHI (2020). Biosafety in Microbiological and Biomedical Laboratories.

